# Sedentary Work and Physiological Markers of Health

**DOI:** 10.3390/ijerph18063230

**Published:** 2021-03-20

**Authors:** Brad Wipfli, Sara Wild, Courtney Donovan, Ginger C. Hanson, Saurabh S. Thosar

**Affiliations:** 1 OHSU-PSU School of Public Health, Portland, OR 97239, USA; bwipfli@pdx.edu (B.W.); thosar@ohsu.edu (S.S.T.); 2 Oregon Institute of Occupational Health Sciences, Oregon Health & Science University, Portland, OR 97239, USA; donovcou@ohsu.edu; 3 School of Nursing, Johns Hopkins University, Baltimore, MD 21218, USA; ghanson4@jhu.edu; 4 School of Nursing, Oregon Health & Science University, Portland, OR 97239, USA; 5 School of Medicine, Oregon Health & Science University, Portland, OR 97239, USA

**Keywords:** sedentary behavior, uninterrupted sitting, occupational health, endothelial function, hemoglobin A1c

## Abstract

The purpose of this study is to examine associations between objectively measured workplace sedentary behavior and physiological markers of health. We hypothesize that increased sedentary time and more frequent bouts of uninterrupted sitting are associated with increased hemoglobin A1c, increased blood pressure, and impaired endothelial function. Call center employees (N = 241) were enrolled from four worksites in the United States. Participants completed a survey and a physical health assessment. Sedentary behavior and sitting/standing time at work were quantified using an accelerometer. Hemoglobin A1c was measured using a finger-prick and portable analyzer. Blood pressure was measured with an automated cuff, and vascular endothelial function was assessed in a subsample of participants (n = 56) using EndoPAT. We analyzed data with two series of ordinary least squares regressions, first to examine relationships between bouts of uninterrupted sitting and physiological outcomes, and second to examine relationships between physical activity and sitting/standing time at work and physiological outcomes. The sample was primarily female, and on average was obese, prehypertensive, and prediabetic. There were no significant relationships between bouts of uninterrupted sitting or physical activity/sitting/standing time at work and physiological outcomes. In a sample that is predominantly sedentary, at risk for cardiovascular disease, and prediabetic, there are no significant associations between workplace sedentary behavior and physiological markers. The lack of associations could be related to either physiological adaptations or ceiling effects in this sample.

## 1. Introduction

Epidemiological evidence shows that sedentary behavior, defined as any waking behavior with energy expenditure ≤1.5 metabolic equivalents, while in a sitting, reclining, or lying posture [1], is related to increased risk of detrimental health outcomes such as cardiovascular disease, diabetes, poor metabolic profile, and all-cause mortality [2,3,4,5,6,7,8,9,10]. Furthermore, these risk factors persist even if one meets the Centers for Disease Control and Prevention’s physical activity recommendations but is sedentary during the majority of waking hours [11]. Recent estimates indicate that adults spend on average >9 h/day in sedentary pursuits, and approximately half of the total sedentary time is spent in bouts ≥30 min [12,13].

Regarding the biological processes that link sedentary behavior to chronic disease, a recent review states that “loss of muscular contractile stimulation induced through prolonged sitting impairs skeletal muscle metabolism of lipids and glucose and that the molecular, genetic, and lipidomic processes through which these responses occur may be both similar to and separate from the pathways activated by engaging in regular exercise” [14] (p. 272). We and others have shown that sedentary behavior and bouts of uninterrupted sitting are related to impairments in physiological functioning such as vascular endothelial function, blood flow, and glucose processing [15,16,17], all of which can lead to chronic diseases. For instance, impaired endothelial function is a strong, early prognostic marker of cardiovascular disease and is impaired even before structural adaptations in the vasculature or any apparent atherosclerosis is detectable [18,19,20,21,22]. Sedentary behavior is a predictor of high hemoglobin A1c levels [23]. A1c is a marker of metabolic dysfunction, and a predictor of type II diabetes [24] and new-onset cardiovascular disease in adults [25]. Additionally, in individuals with impaired glucose processing postprandial blood glucose levels remain high for an extended period of time after prolonged sedentary behavior, resulting in insulin insensitivity and increasing risk for diabetes and cardiovascular disease [26,27,28].

Experimental studies that have discovered the mechanisms by which uninterrupted sitting bouts adversely impact human physiology, and those that tested countermeasures (e.g., breaks in sitting time), have been well-controlled laboratory studies, short in duration (a few hours—two weeks), with small sample sizes [15,17,29,30,31]. The development of chronic disease, however, is an insidious process, and little is known about the physiological effects of habitual sedentary behavior in an applied setting. Laboratory studies have rigorous experimental control and are essential for identifying mechanisms but likely do not reflect real life. Conversely, even though fewer variables can be controlled, applied settings offer the opportunity to understand physiological processes in the ways that people experience them, and the ability to collect data from a larger, more representative sample.

There are myriad domains of everyday life that contribute to sedentary behavior. One of the most significant factors that increases sedentary behavior in the working adult population is the amount of time spent at work [32], where employed adults spend an average of 7.5 hours per day [33]. Sedentary behavior and bouts of uninterrupted sitting at work may compromise the shear mediated protection for the endothelium [21,31] and may also contribute to impaired glucose metabolism [15,30,34]. Despite the potential impact of sedentary workplaces on physiological outcomes, we are aware of only one workplace sedentary behavior study that measured blood glucose outcomes [35]. Furthermore, we are not aware of any large workplace sedentary behavior studies that have measured associations between sedentary behavior and vascular function.

The purpose of this study is to examine associations between workplace sedentary behavior and physiological markers of health in a workplace setting. Call centers are a particularly well-suited setting because the organization of work ties employees to their desks, limiting autonomy for movement, and call center employees report sitting for 83% of work hours [36]. We hypothesize that more time spent in sedentary behavior and more frequent bouts of uninterrupted sitting are related to increased hemoglobin A1c, increased blood pressure, and impaired endothelial function. Furthermore, we hypothesize that there is a dose-response relationship between uninterrupted sitting and physiological markers of health, in which longer durations of uninterrupted sitting are associated with increasingly higher levels of hemoglobin A1c and blood pressure, and greater impairments in endothelial function.

## 2. Methods

### 2.1. Participants

Participants (N = 241) were customer service agents recruited from four call centers in the Western U.S. Three of the organizations provide customer service for utility companies, and the fourth location provides customer service for a health care organization. The job demands and organization of work at each location are similar: participants all had sit/stand desks, and had similar work schedules (day shifts, 8–10 h days with two 15-min breaks and a 30–60 min lunch break), time pressures (maximum of 90–120 s between concluding one call and starting another), and physical environments. Participants were recruited with study advertisements, emails, and announcements from supervisors. Sample characteristics are presented in Table 1. All participants signed written informed consent, and all study procedures were approved by the Oregon Health & Science University Institutional Review Board.

### 2.2. Measures

Upon enrollment, all participants completed an electronic survey, a physical health assessment, a finger-prick measure of hemoglobin A1c, and were set up with an accelerometer. A subsample of volunteer participants (n = 56) completed a measurement of vascular endothelial function. Participants received USD 30 and an additional USD 15 if they completed the vascular endothelial function measurement.

#### 2.2.1. Survey

Participants were emailed an electronic survey via Survey Gizmo and asked to complete it prior to completing their physical health assessment. The survey included measures of demographics, physical activity outside of work [37], and occupational sitting [38].

#### 2.2.2. Physical Health Assessment

Participants completed a physical health assessment that included measures of height, body weight, percent body fat, resting blood pressure, and resting heart rate. Participants started the assessment by sitting for at least 1 to 3 min. They were then asked to put their feet flat on the floor and to not talk while their blood pressure and resting heart rate were measured by a single reading with a digital blood pressure monitor (OMRON, model HEM-907XL). Height was then measured with a stadiometer (seca, model 213) and body weight and percent body fat with a body composition analyzer (Tanita, model TBF-310).

#### 2.2.3. Hemoglobin A1c

The physical health assessment also included a measure of Hemoglobin A1c that was determined using the DCA Vantage Analyzer (Siemens AG, Munich, Germany; precision coefficient of variation ≤2%, NGSP certified method; [39]). Blood samples were obtained by a finger stick from a participant’s third or fourth finger, collected after removing the first drop of blood, and processed immediately.

#### 2.2.4. Accelerometry

Participants were assigned an accelerometer (ActiGraph GT9X Link; ActiGraph LLC, Pensacola, FL, USA) set to a sample frequency of 60 hertz, and asked to wear it on their non-dominant thigh while working for a one-week duration starting the day after the health assessment. For analysis, epoch duration was set to 60 s, and wear time was validated [40]. All wear time data were checked manually and edited to exclude any data outside of work hours or past five full days (defined as 6 or more hours). Physical activity was scored using Freedson Adult (1998) cut points [41], resulting in classifications of Sedentary, Light, Moderate, Vigorous, and Very Vigorous activity. To analyze uninterrupted sitting bouts, we defined bouts as 20+, 30+, 40+, 50+, and 60+ min of consecutive sitting. We calculated frequency counts for uninterrupted sitting bouts of each duration, and total time in bouts of each duration. We then standardized these measures by dividing frequency counts and total time by minutes of validated wear time. In addition, we standardized postural classifications (minutes stepping, sitting/lying, and standing) and physical activity classifications by minutes of validated wear time to calculate percent time spent in each of these activities.

#### 2.2.5. Vascular Endothelial Function

Vascular endothelial function was measured using an estimate of reactive hyperemia (EndoPAT and EndoPAT 2000 software, Itamar Medical Ltd., Caesarea, Israel). EndoPAT uses automated analysis of arterial pulsatile volume changes in the index finger after reactive hyperemia, and is predominantly dependent on nitric oxide bioavailability in healthy participants [42]. EndoScore is the outcome variable, with scores between 2.1–3 indicating optimal functioning, scores between 1.69–2 indicating sub-optimal functioning, and scores 1.68 and below indicating low functioning. Impairment in endothelial function observed using EndoPAT is correlated with impairment in coronary, and brachial artery endothelial function [43,44,45].

Participants were asked to abstain from food for a minimum of four hours, tobacco and caffeine for a minimum of eight hours, and vigorous exercise for a minimum of twenty-four hours before completing the measure, and adherence was measured via self-report. Participants who did not follow protocol instructions, and participants who reported fewer than five hours of sleep the previous night were excluded from analyses. Blood pressure was measured with a digital blood pressure monitor (Omron, model HEM-907XL) and the systolic and diastolic blood pressures were entered into EndoPAT 2000 along with the participant’s age, sex, height, and weight. A blood pressure cuff was put on the participant’s non-dominant arm unless the participant had a need for the cuff to be put on their dominant arm. EndoPAT sensors were placed on the participant’s index fingers. Measurement included three five-minute recording periods: baseline; an occlusion period, where the cuff was rapidly inflated and maintained at 60 mmHg above systolic blood pressure (with a minimum pressure of 200 mmHg and a maximum pressure of 300 mmHg); and post-occlusion after the cuff was rapidly deflated. Automatic analysis was used to calculate the EndoScore.

### 2.3. Analyses

**Figure 1:**
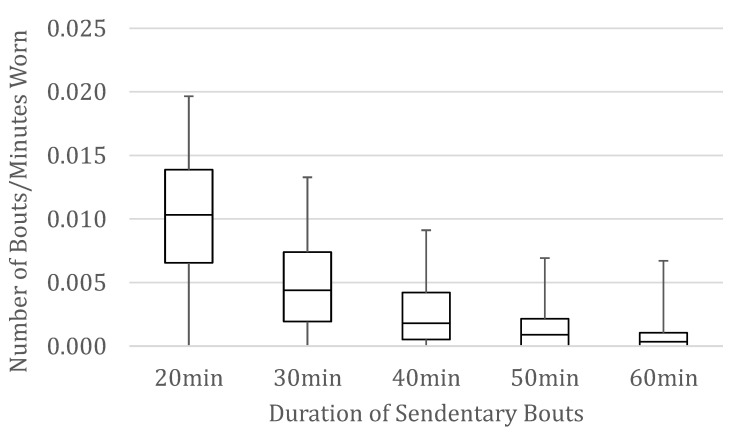
Box and whisker plots of the number of bouts of uninterrupted sitting 20–60 min per minute that the device was worn.

We computed descriptive statistics on the demographic and health characteristics of participants overall and by worksite (see Table 1) and made comparisons between the four worksites. Chi-square analyses were conducted on categorical variables, one-way ANOVAs on normally distributed continuous variables, and Kruskal–Wallis test on variables with skewed distributions. There were significant differences between worksites on several variables, and worksite was therefore included as a covariate in our main analyses. Next, we examined the distributions of standardized bouts of uninterrupted sitting, illustrated with the median interquartile range and lowest and highest scores in Figure 1. The number of 20+-min bouts is normally distributed, and distributions skew more positively for increasingly longer bouts.

The main analyses were a series of ordinary least squares regressions examining the relationship between the number of bouts of uninterrupted sitting of 20+, 30+, 40+, 50+, and 60+ min (separately) with the outcome variables of hemoglobin A1c, EndoScore, and systolic blood pressure. All regression models controlled differences between worksites with dummy coded variables. We ran one set of models with, and one set of models without controlling for physical activity outside of work. Finally, we conducted a second series of regressions to examine relationships between physiological markers (hemoglobin A1c, EndoScore, and systolic blood pressure) and the percentage of work time spent in different classifications of physical activity (sedentary, light, moderate), sitting or lying, stepping, and standing. Each of these regression models contained one predictor and one outcome, controlling for worksite and physical activity outside of work.

## 3. Results

The overall sample was mostly female (77.7%), White (70.2%), and Non-Hispanic (80.3%). The average age of participants was 39.7 years (SD = 11.6), and 19.7% had a college degree. Less than half had children living at home (43.5%), and 18.5% were caregivers for an adult family member. Fourteen percent of participants reported smoking. The average systolic blood pressure was 126 mmHg (18.4) and diastolic blood pressure was 81.4 mmHg (12.3), and 16.4% of participants took medication for high blood pressure. The average BMI was 32.1 kg·m^−2^ (13), body fat 39% (10.3), hemoglobin A1c 5.7 (1.2), EndoScore 2.03 (0.5). On average participants reported participating in 30+ min of moderate to vigorous physical activity 1.25 (2.1) times a week.

**Figure 2:**
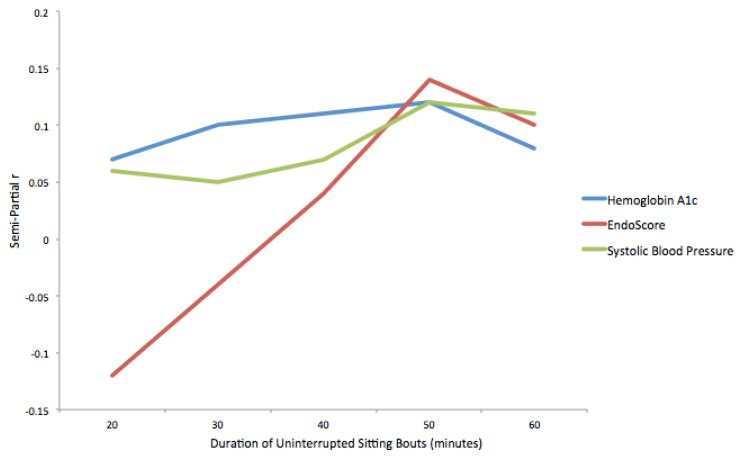
Semi-partial correlation coefficients for relationships between physiological markers and uninterrupted sitting bouts.

The results of regression models using the standardized number of uninterrupted sitting bouts of various durations to predict hemoglobin A1c, EndoScore and systolic blood pressure are presented in Table 2 and Figure 2. The frequency of uninterrupted sitting bouts was not related to hemoglobin A1c, EndoScore, or systolic blood pressure at any bout duration. As the duration of uninterrupted sitting bouts increased from 20 min to 50 min, the effect size, indicated by the semi-partial correlation coefficient, demonstrated a pattern of increasing strength for both hemoglobin A1c (0.07–0.12) and systolic blood pressure (0.06–0.12). A post hoc power analysis was conducted using G*Power 3.1.9.4 [46]. With statistical power set at 0.80 and alpha set at 0.05, a sample size of 540 participants would be needed to detect a semi-partial correlation of 0.12.

**Figure 3:**
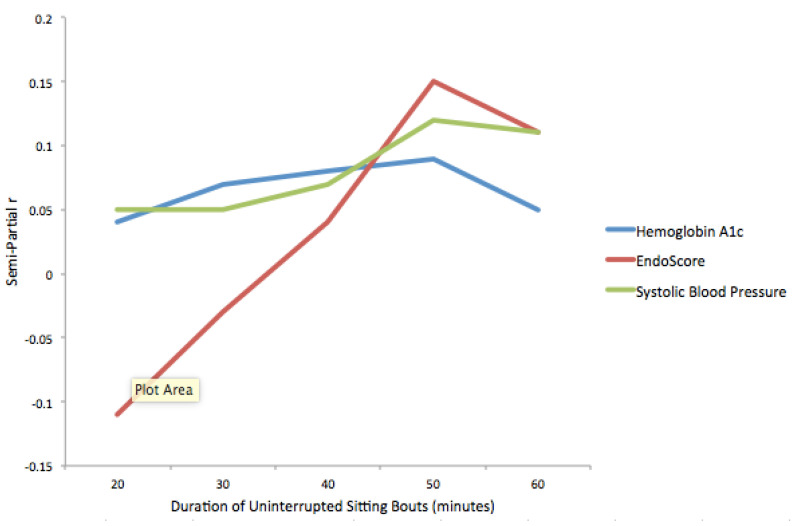
Semi-partial correlation coefficients for relationships between physiological markers and uninterrupted sitting bouts, while controlling for physical activity outside of work.

The results of regression models examining the number of standardized uninterrupted sitting bouts of different durations and physiological markers while accounting for physical activity outside of work are presented in Table 3 and Figure 3. The pattern of results is similar. After controlling for physical activity, no significant relationship was found between the number of uninterrupted sitting bouts of any duration and any of the physiological variables.

Descriptive statistics for percent of work time in different physical activity and postural classifications are presented in Table 4, and the results of regression analyses examining the relationships between these classifications and physiological markers of health are shown in Table 5. None of the classification measures were related to any of the physiological variables after controlling for worksite and physical activity outside of work time. Examining the semi-partial correlations, percent time sitting or lying explained 4% of the variability in EndoScore, with more time spent sitting or lying related to lower EndoScores (indicating worse endothelial functioning). Inversely, percent time standing also explained 4% of the variability in EndoScore, with more time spent standing related to higher EndoScores (indicating better endothelial functioning).

## 4. Discussion

To our knowledge, this is the first study to measure associations between habitual workplace sedentary behavior and physiological variables in an applied occupational setting. Overall, the study did not find support for the hypotheses, indicated by no statistically significant relationships between measures of uninterrupted sitting, sedentary behavior/physical activity, or sitting/standing time and physiological variables. Experimental evidence shows clear associations between sedentary behavior and detrimental physiological outcomes. Similarly, epidemiological studies show clear associations between sedentary behavior and detrimental health outcomes. Yet, in this real-world sample of people with increased baseline cardiovascular and metabolic risks, we failed to support the hypothesis that workplace sedentary behavior is associated with measures of blood pressure, hemoglobin A1c, and vascular endothelial function.

On average, sedentary behavior at work (78% of work hours) and sitting time at work (83% of work hours) in this sample is comparable to data reported in previous studies with call center employees [36]. The sample in this study was also primarily obese and prehypertensive, and while we did not find any associations between sedentary behavior and physiological markers of health, the sample is already at risk for metabolic and cardiovascular diseases. The average EndoScore was 2.03, which is below the optimal range, and the average hemoglobin A1c score was 5.7, which indicates prediabetes. When considering how physiological mechanisms may operate, the high level of sedentary time may contribute to a ceiling effect, wherein the majority of participants have similarly high levels of sedentary behavior and detecting differences on physiological measures is unlikely or impossible. Elevated risk levels in this sample also could have led to a ceiling effect, wherein the majority of participants are already above clinical thresholds, resulting in non-significant associations.

Another possible explanation for the lack of significant associations in this study is physiological adaptations to habitual sedentary behavior. People who have been working in this environment or similar sedentary environments for several years could develop a physiological tolerance, where a larger stimulus (e.g., multiple hours of uninterrupted sitting) is required to impact longer-term physiological markers of health. Such mechanisms may warrant investigation in experimental laboratory studies. Nonetheless, greater frequency of 50-minute bouts trended towards associations with higher hemoglobin A1c and blood pressure, which supports the proposed physiological pathways: uninterrupted prolonged sitting leads to less muscle contraction, lower uptake of glucose, chronically higher fasting blood glucose, and higher hemoglobin A1c [47]; and uninterrupted prolonged sitting leads to lack of muscle contraction, an increase in sympathetic activity to maintain cardiac filling for venous return in the absence of muscle pump, and chronic elevated sympathetic activity leads to higher blood pressure [48,49]. The prevalence of prehypertension and prediabetes in this sample also supports these chronic pathways.

There are several limitations to this study. Foremost is the lack of a control or other reference group. Sedentary behavior was high in all the call centers in this study, which highlights the importance of understanding physiological mechanisms in this group, but also may have contributed to the aforementioned ceiling effects. Future studies in this area should include worksites where sedentary behavior is less pervasive and cardiovascular and metabolic risk levels are lower. As in any applied study, there was limited control over the environment, including participant availability and ability to follow study protocols. We would have liked to measure flow-mediated dilation, which is not feasible in an applied occupational setting. The sample skewed heavily toward females, and, given the significant differences between worksites, differences in workplace environments or cultures could have impacted results. In addition, we used a self-report measure for physical activity outside of work, and asking participants to wear their accelerometers at home may have provided more accurate data. Vascular endothelial functioning was an exploratory measure because feasibility issues limited the number of people who could complete the test, and several people were excluded from these analyses because they did not adhere to the pre-testing protocol. We measured hemoglobin A1c as a marker of metabolic dysfunction and its predictive potential for type II diabetes [24] and new-onset cardiovascular disease in adults [25]. Future worksite studies can further separate out mechanisms by measuring postprandial glucose, fasting glucose, and measures of insulin resistance. The data presented here may help inform power analyses in future studies.

## 5. Conclusions

This study addresses a gap in sedentary behavior research by investigating how objectively measured workplace sedentary behavior is related to physiological markers of health in an applied setting. In a sample that is predominantly sedentary, at risk for cardiovascular disease, and prediabetic, there are no significant associations between workplace sedentary behavior and systolic blood pressure, hemoglobin A1c, or endothelial function. However, trends in the results support existing proposed physiological pathways for sedentary behavior and chronic disease. The lack of associations could be related to either physiological adaptations to habitual sedentary behavior or ceiling effects in this sample, which could be addressed in future research. These study results provide important information for moving the field of sedentary behavior physiology research forward in an applied setting by informing the size and composition of samples for future studies. It would be beneficial to study the association between sedentary behavior and physiological markers of health in a sample that is new to sedentary work, at lower risk for chronic disease, or over a longer period of time.

## Figures and Tables

**Table 1:** Differences Between Groups on Demographics and Physiological Variables.

		Worksite				
	Total N = 241	1 N = 55	2 N = 75	3 N = 68	4 N = 43	*p*
**Sex(male)**	**22.3%**	**35.2%**	**32.9%**	**8.8%**	**9.3%**	**<0.001**
**Race (white)**	**70.2%**	**48.1%**	**78.1%**	**72.1%**	**81.4%**	**0.001**
Race						NA
White	64.7%	43.6%	70.7%	69.1%	74.4%	
Black	9.5%	20.0%	1.3%	14.7%	2.3%	
Native American	2.1%	5.5%	2.7%	0.0%	0.0%	
Asian	1.7%	1.8%	0.0%	2.9%	2.3%	
Pacific Islanders	2.5%	1.8%	2.7%	0.0%	7.0%	
Other	14.1%	21.8%	16.0%	10.3%	7.0%	
Multi-Racial	5.4%	5.5%	6.7%	2.9%	7.0%	
**Hispanic**	**19.7%**	**24.1%**	**28.8%**	**10.3%**	**14.0%**	**0.028**
**Education**						**0.012**
**HS or Less**	**31.5%**	**22.2%**	**45.2%**	**23.5%**	**32.6%**	
**Some College**	**48.7%**	**59.3%**	**45.2%**	**50.0%**	**39.5%**	
**Bachelors or Higher**	**19.7%**	**18.5%**	**9.6%**	**26.5%**	**27.9%**	
Children	43.5%	41.5%	54.8%	36.8%	37.2%	0.121
Caregiver for Adult	18.5%	16.7%	24.7%	11.8%	20.9%	0.243
**Smoke**	**14.3%**	**14.8%**	**24.7%**	**8.8%**	**4.7%**	**0.010**
Blood Pressure Medication	16.4%	20.4%	12.3%	11.8%	25.6%	0.157
	M(SD)	M(SD)	M(SD)	M(SD)	M(SD)	
**Age**	**39.72** **(11.64)**	**40.34** **(11.18)**	**35.45** **(9.98) ^a,b^**	**42.01** **(11.63) ^a^**	**42.59** **(13.10) ^b^**	**0.001**
**Diastolic blood pressure**	**81.43** **(12.27)**	**87.13** **(13.02) ^e,f,g^**	**81.19** **(12.22) ^e^**	**78.04** **(10.50) ^f^**	**79.93** **(11.85) ^g^**	**<0.001**
**Systolic blood pressure**	**126.10** **(18.35)**	**133.35** **(22.73) ^c,d^**	**123.39** **(16.43) ^c^**	**123.31** **(15.93) ^d^**	**125.98** **(16.88)**	**0.007**
**Percent body fat**	**38.90** **(10.25)**	**37.08** **(10.69) ^h^**	**37.50** **(10.83) ^i^**	**39.27** **(10.20)**	**43.01** **(7.49) ^h,i^**	**0.019**
Hemoglobin A1c	5.74 (1.20)	6.01 (1.63)	5.66 (1.21)	5.54 (0.63)	5.84 (1.15)	0.144
EndoScore	2.03 (0.49)	1.86 (0.41)	2.17 (0.50)	2.02 (0.50)	2.07 (0.53)	0.408
	Median (IQR)	Median (IQR)	Median (IQR)	Median (IQR)	Median (IQR)	
Physical activity	1.25 (2.06)	1.50 (2.13)	1.00 (1.75)	1.25 (2.25)	1.00 (2.50)	0.442
BMI	32.10 (12.90)	31.90 (11.40)	31.70 (11.50)	30.30 (12.35)	34.60 (16.20)	0.259

Note. Chi-square analyses were conducted on categorical data, one-way ANOVAs on normally distributed continuous variables, and Kruskal–Wallis test on variables with a skewed distribution. Means with the same subscript are statistically different at a 0.05 level. EndoScore optimal range = 2.1–3, below optimal = 1.69–2, low = 1.68 and below. Physical activity = days of 30+ min of moderate to vigorous physical activity per week Variables with significant differences are in bold. The superscript letters show significant differences between worksites for certain variables.

**Table 2:** Regression Coefficients for Frequency of Uninterrupted Sitting Bouts and Physiological Markers.

**Hemoglobin A1c**	**N**	**b**	***p***	**95% CI**	**Semi-Partial r**
Frequency of 20+ min bouts	236	17.42	0.305	−15.97–50.81	0.07
Frequency of 30+ min bouts	236	37.74	0.111	−8.72–84.21	0.10
Frequency of 40+ min bouts	236	52.09	0.106	−11.22–115.40	0.11
Frequency of 50+ min bouts	236	83.10	0.070	−6.94–173.15	0.12
Frequency of 60+ min bouts	236	78.14	0.229	−49.41–205.68	0.08
**EndoScore**	**N**	**b**	***p***	**95% CI**	**Semi-Partial r**
Frequency of 20+ min bouts	56	−12.00	0.393	15.96–−0.12	−0.12
Frequency of 30+ min bouts	56	−5.59	0.782	−45.85–34.68	−0.04
Frequency of 40+ min bouts	56	8.65	0.765	−49.053–66.359	0.04
Frequency of 50+ min bouts	56	37.81	0.301	−34.84–110.47	0.14
Frequency of 60+ min bouts	56	34.72	0.476	−62.413–131.844	0.10
**Systolic Blood Pressure**	**N**	**B**	***p***	**95% CI**	**Semi-Partial r**
Frequency of 20+ min bouts	237	220.45	0.388	−281.71–722.61	0.06
Frequency of 30+ min bouts	237	299.22	0.401	−400.92–999.36	0.05
Frequency of 40+ min bouts	237	562.95	0.245	−388.90–1514.11	0.07
Frequency of 50+ min bouts	237	1266.54	0.066	−84.30–2617.37	0.12
Frequency of 60+ min bouts	237	1628.13	0.093	−273.54–3529.78	0.11

Note. Analyses controlled for worksite by including dummy variables.

**Table 3:** Regression Coefficients for Frequency of Uninterrupted Sitting Bouts and Physiological Markers, Controlling for Physical Activity.

**Hemoglobin A1c**	**N**	**b**	***p***	**95% CI**	**Semi-Partial r**
Frequency of 20+ min bouts	234	9.96	0.550	−22.84–42.77	0.04
Frequency of 30+ min bouts	234	26.40	0.258	−19.47–72.27	0.07
Frequency of 40+ min bouts	234	40.47	0.203	−21.95–102.88	0.08
Frequency of 50+ min bouts	234	66.29	0.141	−22.13–154.71	0.09
Frequency of 60+ min bouts	234	52.72	0.406	−72.13–177.57	0.05
**EndoScore**	**N**	**b**	***p***	**95% CI**	**Semi-Partial r**
Frequency of 20+ min bouts	56	−11.25	0.427	−39.42–16.93	−0.11
Frequency of 30+ min bouts	56	−4.70	0.817	−45.21–35.82	−0.03
Frequency of 40+ min bouts	56	9.37	0.747	−48.61–67.34	0.04
Frequency of 50+ min bouts	56	40.55	0.271	−32.58–113.67	0.15
Frequency of 60+ min bouts	56	38.42	0.434	−59.41–136.26	0.11
**Systolic Blood Pressure**	**N**	**b**	***p***	**95% CI**	**Semi-Partial r**
Frequency of 20+ min bouts	235	207.14	0.427	−306.01–720.30	0.05
Frequency of 30+ min bouts	235	281.89	0.440	−435.76–999.54	0.05
Frequency of 40+ min bouts	235	553.74	0.264	−420.40–1527.88	0.07
Frequency of 50+ min bouts	235	1262.39	0.072	−115.48–2640.27	0.12
Frequency of 60+ min bouts	235	1611.05	0.102	−323.07–3545.17	0.11

Note. Analyses controlled for worksite and physical activity outside of work.

**Table 4:** Descriptive Statistics for Physical Activity and Postural Classifications.

Variable	Mean (SD)
% Work Hours Sitting or Lying	83.07 (12.91)
% Work Hours Standing	13.22 (12.29)
% Work Hours Stepping	3.70 (3.18)
% Work Hours in Sedentary Activity	77.81 (8.89)
% Work Hours in Light Activity	19.35 (8.29)
% Work Hours in Moderate Activity	2.67 (1.79)

**Table 5:** Regression Coefficients for Work Activity Variables and Physiological Markers.

**Hemoglobin A1c**	**N**	**b**	***p***	**95% CI**	**Semi-Partial r**
% Work Hours Sitting or Lying	234	0.00	0.819	−0.01–0.01	0.02
% Work Hours Standing	234	0.00	0.948	−0.01–0.01	−0.00
% Work Hours Stepping	234	0.03	0.248	−0.08–0.02	−0.07
% Work Hours in Sedentary Activity	234	0.01	0.566	−0.01–0.02	0.04
% Work Hours in Light Activity	234	−0.01	0.565	−0.024–0.013	−0.04
% Work Hours in Moderate Activity	234	−0.00	0.989	−0.09–0.09	−0.00
**EndoScore**	**N**	**b**	***p***	**95% CI**	**Semi-Partial r**
% Work Hours Sitting or Lying	56	−0.01	0.112	−0.02–0.00	−0.21
% Work Hours Standing	56	0.01	0.136	−0.00–0.15	0.20
% Work Hours Stepping	56	0.03	0.435	−0.04–0.09	0.11
% Work Hours in Sedentary Activity	56	−0.01	0.409	−0.02–0.01	−0.11
% Work Hours in Light Activity	56	0.01	0.333	−0.01–0.02	0.13
% Work Hours in Moderate Activity	56	−0.01	0.834	−0.11–0.09	−0.03
**Systolic Blood Pressure**	**N**	**b**	***p***	**95% CI**	**Semi-Partial r**
% Work Hours Sitting or Lying	235	−0.15	0.127	−0.33–0.04	−0.10
% Work Hours Standing	235	0.15	0.139	−0.05–0.341	0.10
% Work Hours Stepping	235	0.73	0.053	−0.01–1.46	0.12
% Work Hours in Sedentary Activity	235	0.05	0.707	−0.22–0.33	0.02
% Work Hours in Light Activity	235	−0.11	0.442	−0.41–0.18	−0.05
% Work Hours in Moderate Activity	235	0.67	0.335	−0.70–2.03	0.06

Note. Analyses controlled for worksite and physical activity outside of work.

## Data Availability

Data for this manuscript may be made available by request at the conclusion of the study.
